# Design, Synthesis and Evaluation of Dual-Modality Glyco-Nanoparticles for Tumor Imaging

**DOI:** 10.3390/molecules18066425

**Published:** 2013-05-30

**Authors:** Hua Zhu, Jun Zhao, Xinfeng Lin, Ye Hong, Chun Li, Zhi Yang

**Affiliations:** 1 Key Laboratory of Carcinogenesis and Translational Research (Ministry of Education), Department of Nuclear Medicine, Peking University Cancer Hospital & Institute, Beijing 100142, China; 2 Department of Experimental Diagnostic Imaging, Unit 59, the University of Texas MD Anderson Cancer Center,1515 Holcombe Boulevard, Houston, TX 77030, USA; 3 China Institute of Atomic Energy, Beijing 102413, China

**Keywords:** indium-111, d-Glucosamine, core-cross-linked polymeric micelles, glyco-nanoparticles, tumor imaging agent

## Abstract

d-Glucosamine (DG) was conjugated to a core-cross linked polymeric micelle (CCPM) system equipped with both a near-infrared fluorophore (NIRF) and a gamma emitter (^111^In). The resultant nano-scale tumor-targeting imaging tracer, ^111^In-DG-NIRF-CCPM, selectively accumulated in a human epithelial carcinoma A-431 xenograft model in mice. At 24 hrs post injection, the tumor uptake was 2.62 ± 0.80 % of the injected dose per gram of tissue (%ID/g). Tumors were clearly delineated in both single-photon emission computed tomography (SPECT) and optical imaging. The results suggest that the prepared imaging tracer is a promising agent for tumor diagnosis.

## 1. Introduction

Optical imaging is a relatively new molecular imaging modality that offers non-invasive, real-time and high-sensitivity imaging of fluorophores embedded in diseased tissues [1,2]. There has been an increased interest in the use of polymeric micelles to deliver tumor-imaging tracers, because polymeric micelles with adequate size can accumulate within tumor tissue due to the enhanced permeation and retention (EPR) effect [3,4]. Specifically, polymeric micelles containing NIRF compounds have been extensively explored for non-invasive molecular imaging [5,6]. Previously, we reported the synthesis of core-cross linked polymeric micelles (CCPM) containing a Cy-7-like NIRF dye as well as a gamma-emitter ^111^Indium (T_1/2_ = 2.83 d, 0.171 MeV (90%), 0.245 MeV (94%)). CCPM system exhibited prolonged blood half-life and enhanced uptake in tumor [7,8]. In this study, we investigated whether the surface of CCPM can be further modified with sugar molecules to improve its pharmacokinetics profile and minimize its non-tumor uptake [9]. 

Carbohydrate-mediated molecular recognitions have been applied to nano-medicine to improve drug delivery efficiency of nano-carriers [10,11]. Glyco-nanotechnology, as an innovative medicine, has been extensively used in basic and clinical research [12,13]. d-Glucosamine (DG) is an attractive scaffold as a glucosyl ligand. We recently reported the synthesis of ^68^Ga- and ^111^In-labeled DG for the positron emission tomography (PET) and SPECT imaging of tumors [14,15,16]. Those studies have showed that DG attached to a bulky moiety still preserved its biological functions. In this study, we conjugated DG to the CCPM surface via a succinate linker, and investigated its performances regarding the tumor targeting, *in vitro* and *in vivo* stability, biodistribution, and dual gamma and NIRF optical imaging. We found that DG-CCPM had excellent stability, could be taken up by tumor cells, and was visualizable by both gamma and optical imaging modalities. Further studies are needed to improve the drug targeting efficiency with dual-labeled CCPM for molecular imaging.

## 2. Results and Discussion

### 2.1. Synthesis and Characterization of CCPM Nanoparticles

DG-COOH was synthesized by the amidation of glucosamine to succinic anhydride. The identity of this product was confirmed by negative mode electrospray mass spectrometry (Agilent LC/MSDTOF), *m/z* = 278.1027 for [M−H]^−^(C_10_H_16_NO_8_, calculated [M−H]^−^ 278.0876);IR (KBr) *ν*: 3048, 2932, 2840, 1518, 1062, 928 cm^−1^; ^1^H-NMR (D_2_O, 300 MHz) *δ*: 7.81 (s, 1H, NH), 5.95 (m, 1H), 4.02 (m, 1H), 3.55 (d, *J* = 2.0 Hz, 2H), 3.30–3.22 (m, 2H), 2.69 (d, *J* = 2.0 Hz, 2H), 2.55 (d, *J* = 2.0 Hz, 2H), 2.3–2.2 (m, 4H, -OH).

**Figure 1:**
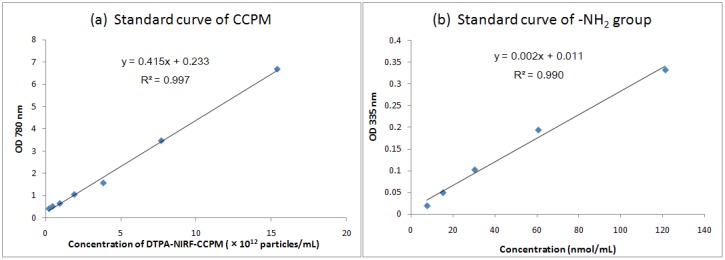
Standard Curve of (**a**) OD 780 nm absorption of DTPA-NIRF-CCPM. (**b**) OD 335 nm absorption of amines.

DTPA was conjugated to CCPM by simply mixing NCS-Bz-DTPA and CCPM under nitrogen overnight. Unreacted NCS-Bz-DTPA was removed by centrifugal filtration or by PD-10 column chromatography. About 70% of the NCS-Bz-DTPA was attached to the NIRF-CCPMs. Next, DG-COOH was coupled to CCPM using1-ethyl-3-(3-dimethylaminopropyl)carbodiimide hydrochloride (EDC) as the coupling agent to form ester linkage. A UV-Vis spectrophotometer was used to measure the absorbance (optical density, OD) at 780 nm or 335 nm; which was converted into a concentration using the Beer-Lambert relation (path-length = 1mm). The standard curve of DTPA-NIRF-CCPM generated at 780 nm was used to determine the concentration of CCPM [Figure 1(a)]. The standard curve generated at 335 nm after treatment with 2,4,6-trinitrobenzenessulfonate (TNBS) was used to quantify the amount of amines on the surface of CCPM [Figure 1(b)] [17,18,19].

The DTPA-NIRF-CCPM-DG (DG-CCPM) nanoparticles were characterized with regard to composition, size, surface charge and optical properties. As shown in Table 1. DG-CCPM nanoparticles had approximately 19 DTPA and 15 DG molecules per nanoparticle. The volume average size of DTPA-NIRF-CCPM was 24 ± 8.9 nm, determined by dynamic light scattering. Prior to conjugation, the amine-abundant CCPM was weakly positively charged. After DTPA and DG conjugation, however, the surface potential changed to slightly negative, indicating the successful conjugation.

**Table 1:** Summary of the physicochemical properties of CCPM.

Specification	NIRF-CCPM	DTPA-NIRF-CCPM	DG-CCPM
Particles (nm) ^a^	24 ± 8.9	24 ± 8.9	24 ± 8.9
Zeta potential (mV) ^b^	+1.2	−5.83 ± 2.40	−5.83 ± 1.08
Dyes/Particle ^c^	21	21	21
DTPA/Particle ^d^	0	19	19
Amines/Particle ^e^	180	160	145
Concentration ^f^	1.54 × 10^15^	7 × 10^14^	3.4 × 10^14^
DG/Particle ^g^	N/A	N/A	15

^a^. Volume-average mean diameter measured by dynamic light scattering. ^b^. Zeta potential measured in phosphate-buffered saline at pH 7.4. ^c^. The value was estimated assuming all dye molecules were incorporated into the nanoparticles. ^d^. DTPA loading per particle; estimated on the basis of amount of DTPA used and of conjugation efficiency as measured by capillary electrophoresis. ^e^. TNBS (OD 335 nm) was used to measure the primary amine groups in each particle. ^f^. Determined by absorption at 780 nm. ^g^. Determined by subtracting the number of amines per nanoparticle after conjugation from the number of amines per nanoparticle before DG conjugation. N/A: not applicable.

### 2.2. Radiolabeling of the Nanoparticles

**Figure 2:**
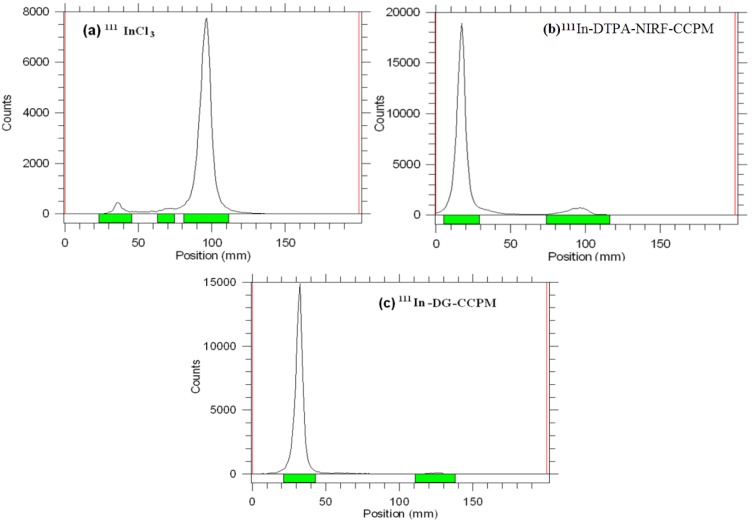
Radio-TLC analyses of (**a**) ^111^InCl_3_. (**b**) ^111^In-DTPA-NIRF-CCPM. (**c**) ^111^In-DG-CCPM.

Radiolabeling of the nanoparticles was accomplished through incubation of ^111^InCl_3_ with DTPA-NIRF-CCPM or DG-CCPM in 0.1 M NaOAc (pH 5.2) at room temperature, respectively. Under such conditions, a labeling efficiency of greater than 96% was achieved without further purification. As indicated by radio-TLC, most radioactivity was associated with ^111^In-labeled DTPA-NIRF-CCPM or DG-CCPM nanoparticles (*R*_f_~ 0.0), with less than 5% detectable free ^111^InCl_3_ (*R*_f_ ~ 0.9) (Figure 2).

### 2.3. Microscopic Imaging of DTPA-NIRF-CCPM-DG

**Figure 3:**
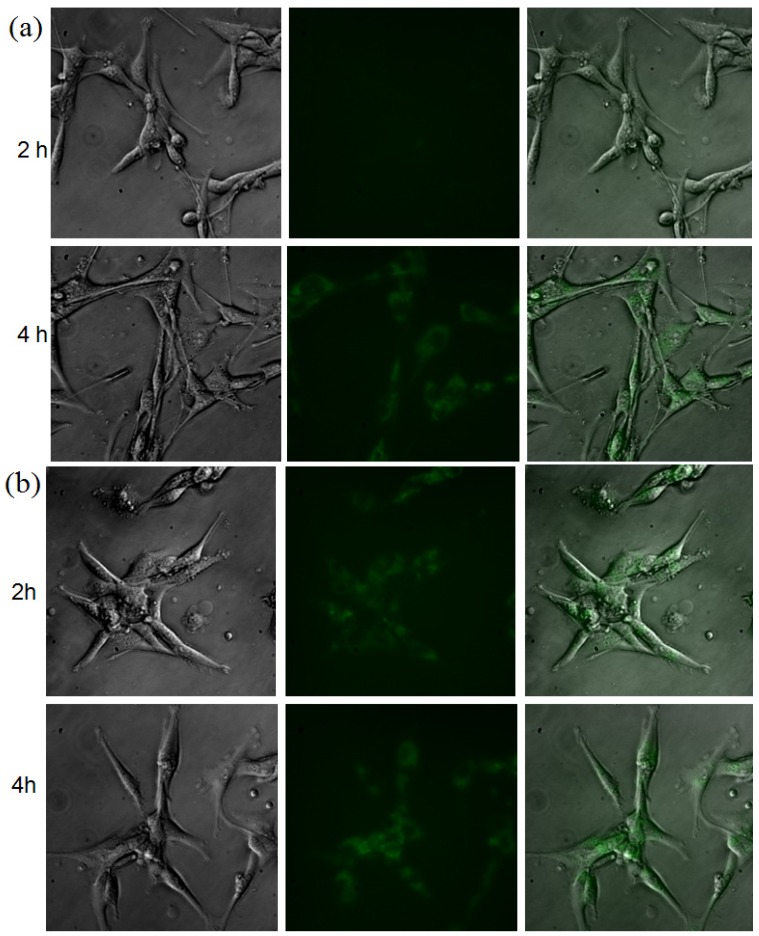
Representative microphotographs of A431 cells after incubation with (**a**) NIRF-CCPM and (**b**) DG-CCPM under (left) bright light, (middle) fluorescence microscope at 750 nm, and (right) overlap of bright light and fluorescence images. Original magnification × 20 object.

The binding of NIRF-CCPM and DG-CCPM to A431 cells was studied by fluorescence microscopy (Figure 3). At two hours after incubation, NIRF-CCPM did not uptake in these cells. However, at 4 hours after incubation, uptake of NIRF-CCPM in cells was clearly visualized. In comparison, uptake of DG-CCPM in A431 cells were observed at 2-hour time point, and the fluorescent signal intensity appeared to have increased over from 2 to 4 h (Figure 3). The data indicated that both NIRF-CCPM and DG-CCPM could be internalized over time. Cell uptake of NIRF-CCPM could be attributed to nonspecific phagocytic activity of tumor cells. Many studies have demonstrated that ^18^F-FDG and its fluorescent analogs are substrates of glucose transporters (GLUTs) [20]. Further studies are needed to establish whether uptakes of DG-CCPM in A431 cells are mediated through GLUTs.

### 2.4. Biodistribution in Tumor Bearing mice

**Figure 4:**
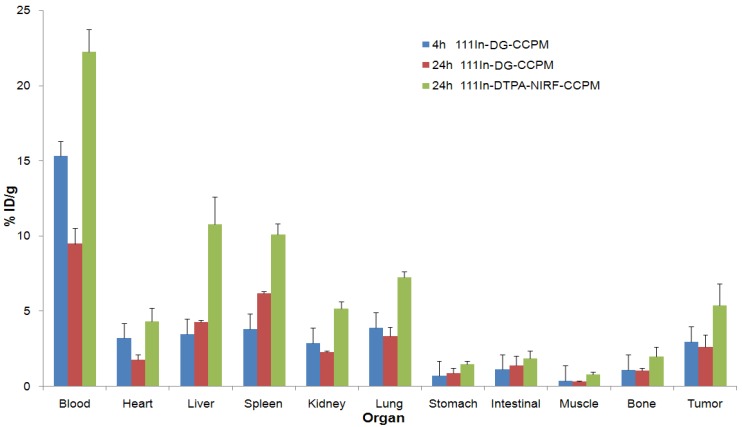
Biodistribution studies of ^111^In labeled CCPM in nude mice bearing A 431 tumors at 4 h and 24 h.

Biodistribution studies of ^111^In-DG-CCPM and ^111^In-DTPA-NIRF-CCPM were carried out in mice bearing subcutaneous A431 tumor. As shown in Figure 4, the percentage of injected dose per gram of tissue (%ID/g) at 24 h after intravenous injection in the liver and the spleen were 4.27 ± 0.15% and 6.2 ± 0.14% for ^111^In-DG-CCPM, and 10.79 ± 1.81% and 10.1 ± 0.74% for ^111^In-DTPA-NIRF-CCPM, respectively. ^111^In-DG-CCPM had significantly lower spleen uptake than that of the ^111^In-DTPA-NIRF-CCPM (*p* = 0*.*0097). On the other hand, ^111^In-DTPA-NIRF-CCPM had significantly higher activity in the blood (*p* = 0*.*0098) and the lung (*p* = 0*.*0067). ^111^In-DTPA-NIRF-CCPM activity was also significantly higher in the tumor (*p* = 0*.*046). The higher activity observed with ^111^In-DTPA-NIRF-CCPM as compared to ^111^In-DG-CCPM in the liver, spleen, lung, and tumor may be attributed to higher blood pool activity of ^111^In-DTPA-NIRF-CCPM.

**Figure 5:**
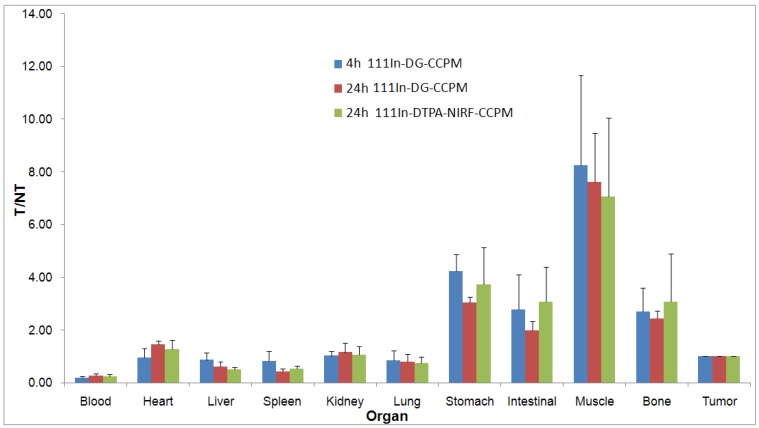
Comparison of tumor-to-normal tissue uptake ratio (T/NT) between ^111^In-DTPA-NIRF-CCPM and ^111^In-DG-CCPM in nude mice bearing A 431 tumors at 4 h and 24 h.

However, the distribution patterns for each particle were similar, with the liver and the spleen being the major organs taking up the radio-labeled glyco-nanoparticles. Figure 5 compares the tumor-to-normal tissue (T/NT) uptake ratio of ^111^In-DG-CCPM and ^111^In-DTPA-NIRF-CCPM. ^111^In-DTPA-NIRF-CCPM did not show significantly higher T/NT ratios compared with ^111^In-DG-CCPM at 24 h post injection.

### 2.5. *In Vivo* Gamma Imaging

**Figure 6:**
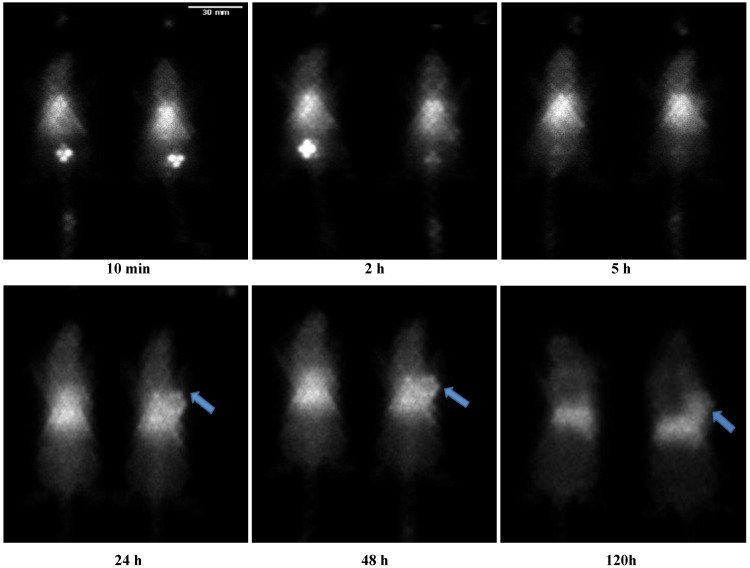
Representative gamma images of A 431 bearing nude mice at various times (left: ^111^In-DTPA-NIRF-CCPM; right: ^111^In-DG-CCPM). Arrows indicate the presence of tumors.

Figure 6 shows the representative gamma images obtained at various time points after injection of ^111^In-DTPA-NIRF-CCPM (300µCi/2.6 × 10^14^particles/mouse) and ^111^In-DG-CCPM (300µCi/4 × 10^13^ particles/mouse). Both nanoparticles exhibited sustained blood pool activity, prominent and persistent accumulation in the liver and the spleen for up to 120 h, and gradual accumulation in the tumor. The cardiac area was clearly seen up to 48 h post injection, reflecting a long blood circulation time for CCPMs. Uptake in the tumor was clearly visualized by 120 h post injection with *γ*-scintigraphy. Tumors in the mice received intravenous injection of ^111^In-DG-CCPM, but not in mice received ^111^In-DTPA-NIRF-CCPM, were visualized at later time points (24–72 h post injection) (Figure 6).

### 2.6. *In vivo* NIRF Imaging

**Figure 7:**
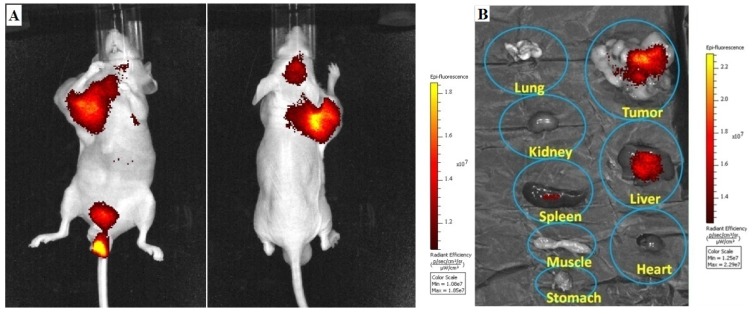
(**A**) Representative NIRF images of A 431 bearing nude mice at 120 h after intravenous injection of ^111^In-DG-CCPM. (**B**) Representative NIRF images of various excised tissues from a mouse received ^111^In-DG-CCPM at the end of the imaging sessions (120 h post-injection).

The biodistribution of ^111^In-DG-CCPM (4 × 10^13^ particles) in A431-bearing mice was also determined by in and *ex vivo* analysis of the fluorescence signal intensities of excised tissues (Figure 7A,B). Accumulation of ^111^In-DG-CCPM nanoparticles in the tumor could be readily visualized with optical imaging (Figure 7A). In consistent with gamma imaging finding, the highest activity was found in the tumor, the liver and the spleen (Figure 7B). This indicates that glyco-CCPM nanoparticles displayed a strong and persistent fluorescence signal *in vivo*. Clearly, nuclear imaging and NIRF optical imaging techniques provided complementary information. 

Although our biodistribution data do not support active targeting of DG-CCPM to A431 tumors at 24 h after injection, the imaging data appears to show better tumor delineation with ^111^In-DG-CCPM than with ^111^In-DTPA-NIRF-CCPM at 24–72 h post injection. One possible explanation is the relatively small size of DG (MW = 278 Dalton), which could be easily masked by much larger PEG chains on nanoparticles’ surface. Our finding in the current study differs from that of an earlier report which did validate active targeting when the same NIRF-CCPM was used but with a different targeting ligand. In that study, the targeting ligand is annexin V, which has a molecular weight of ~35,000 Dalton [21]. Larger ligand relative to CCPM may be less prone to the masking effect of PEG. Another possibility is heterogeneous intratumoral distribution of ^111^In-DG-CCPM, which could lead to insignificant change in tumor uptake value because the radioactivity counting method averages out contribution from the entire tumor mass. Although our data do not conclusively support active targeting with ^111^In-DG-CCPM, these data provide valuable insight that may help design better ligand-CCPM conjugate for targeted nanoparticle delivery.

## 3. Experimental

### 3.1. General

D-Glucosamine (DG), sodium acetate (NaOAc), phosphate-buffered saline (PBS), N-methyl-morpholine (NMM), succinic anhydride (SA) was purchased from Sigma-Aldrich (St. Louis, MO, USA). 2-(4-Isothiocyanatobenzyl)-diethylenetriamine-pentaacetic acid (NCS-Bz-DTPA) was purchased from Macrocyclics (Dallas, TX, USA). ^111^InCl_3_ was obtained from Iso-Tex Diagnostics (Houston, TX, USA). Other commercial chemicals were purchased from VWR International (San Diego, CA, USA). All reagents were analyzed before use. Particle size was measured using dynamic light scattering with a Malvern ZetaSizer Nano-ZS system (Malvern Instruments Ltd., Worcestershire, UK). Zeta potential was determined using a ZetaPlus Analyzer (Brookhaven Instruments Corp., Holtsville, NY, USA).

^1^H-NMR spectra were recorded on a Varian XL-300 spectrometer (Varian, Inc., Palo Alto, CA, USA) operating at 300 MHz with tetramethylsilane (TMS) as an internal standard. The samples were separated using two 7.5 × 300 mm PLgel mixed-Columns’ (Polymer Laboratories, Amherst, MA, USA) eluted with1,1,1,3,3,3-hexafluoroisopropanol containing 0.01 M tetraethyl ammonium nitrate at a flow rate of 1.0 mL/min. Instant thin-layer chromatograms were recorded using a Bioscan scanner (Bioscan, IAR-2000, Washington, DC, USA). Radioactivity was counted using an automated gamma scintillation counter (Perkin Elmer, 1470-002, Boston, MA, USA). High performance liquid chromatography (HPLC) was performed using a Dionex P680 system equipped with a tunable absorption detector and a PDA-100 photodiode-array detector using a Hypersil BDS C-18 reversed-phase column (5 μm, 250 mm × 4.6 mm). The column was eluted with 0.1%TFA in H_2_O (solvent A) and 0.1%TFA in acetonitrile (solvent B). The eluting conditions were as follow: Solvent B: 0%~10% from 0–5 min, Solvent B: 10%~50% from 5–8 min, Solvent B: 50%~80% from 8–10 min, and Solvent B: 80%~10% from10–12 min.

### 3.2. Synthesis and Characterization of DG-CCPM

#### 3.2.1. Synthesis of d-Glucosamine-NH-SA

d-Glucosamine-NH-SA (DG-COOH) was synthesized by treating commercially available d-Glucosamine hydrochloride with succinic anhydride using NMM as a base. Briefly, succinic anhydride (1.0 mmol) and NMM (0.2 mL) were added to a solution of d-Glucosamine hydrochloride (1.0 mmol) in 10 mL of dimethylformamide (DMF)/H_2_O (9/1, v/v). The mixture was stirred at ambient temperature for 12 h. After evaporation of the solvents under vacuum, the residue was washed with ether. Residue was dissolved in water and purified by HPLC to yield a white powder.

#### 3.2.2. Conjugation of DG-COOH to CCPM

**Scheme 1:**
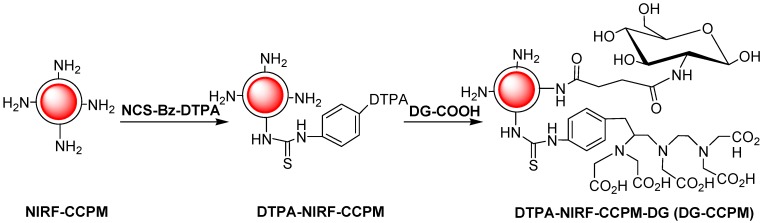
Synthetic strategy of DG-CCPM.

DTPA-CCPM was synthesized according to previously reported procedures with some modification (Scheme 1) [7]. An aqueous solution of CCPM nanoparticles (2 mL, 2 mg dry weight/mL of water) was placed in an amber vial. The pH of the solution was adjusted to 7.5 using 2% sodium bicarbonate solution. NCS-Bz-DTPA (0.013 mg, 1.54 × 10^−3^ M in PBS) was added to the vial. The reaction was stirred under nitrogen overnight. Unreacted NCS-Bz-DTPA was removed by centrifugal filtration using a membrane with a molecular weight cutoff of 30, 000. The unreacted NCS-Bz-DTPA was quantified by capillary electrophoresis. Approximately 70% of the DTPA used was attached to the particles.

To couple DG-COOH to the primary amines on the surface of CCPM, DG-COOH (17.0 mg in 2.0 mL 0.1 M PBS) was allowed to react with EDC (50 µL 2.232 mg/mL) at room temperature (RT) for 30 min. Then 0.5 mL CCPM (1.54 × 10^15^ particles/mL) was added and the reaction mixture was stirred at RT overnight. The molar ration between amine groups in CCPM and DG-COOH was 1:3. The reaction mixture was purified with PD-10 column, which was eluted with 0.01 M PBS (pH7.4). The amount of amines before and after treatment with DG-COOH was were determined by 2,4,6-trinitrobenzenessulfonate (TNBS) assay.

#### 3.2.3. Standard Curves of DTPA-NIRF-CCPM

The concentration of DTPA-NIRF-CCPM nanoparticles was quantified by comparing UV-Vis absorption at 780 nm with a standard curve. DTPA-NIRF-CCPM in 0.01 M pH 7.4 PBS was serially diluted with PBS to obtain concentrations ranging from 15.4 × 10^12^/mL to 2.4 × 10^11^/mL, and their UV/Vis spectra measured with a UV-Vis spectrometer. 

2,4,6-Trinitrobenzenesulfonate (TNBS) was used to quantify the number of primary amines in CCPM. Briefly, 0.5 mL TNBS in 0.1 M pH 8.5 sodium bicarbonate (0.01%, w/v) was added to each 1 mL solution of phenylalanine standard in NaHCO_3_ buffer ranging from 2 to 20 μg/mL. The mixtures were then incubate at 37 °C for 2 h, followed by addition of 0.5 mL of 10% sodium dodecyl sulfate (SDS) and 0.25 mL of 1 M HCl to each sample. The absorbance of the solutions was measured with UV/Vis spectrometer at 335 nm. 

### 3.3. Radiolabeling

**Scheme 2:**
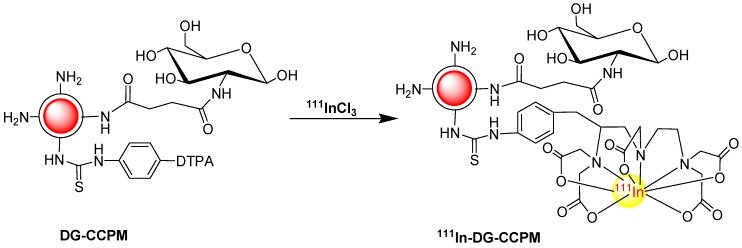
^111^InCl_3_radio-labeling DG-CCPM.

The radiolabeling of DG-CCPM was shown in Scheme 2. Into 0.2 mL aqueous solution of DG-CCPM containing 6.5 × 10^13^ particles was added 10 µL 1M pH 5.2 NaOAc buffer, 40Μl ^111^InCl_3_(520µCi). 

The mixture was allowed to stand at RT for 30 min. Radiolabeled nanoparticles were analyzed using a thin layer chromatography (radio-TLC) system. The Radio-TLC strips were developed with 0.01 M PBS (pH 7.4) containing 4 mM EDTA and quantified using a Bioscan IAR-2000 TLC Imaging Scanner (Washington, DC, USA). Free ^111^In^3+^ moved to the solvent front (Rf~0.9), and the nanoparticles remained at the original (Rf~0.0). The labeling efficiency for DG-CCPM was >96%. The radiochemical purity was > 96%. DTPA-NIRF-CCPM was labeled with^111^Inas a control using similar procedures.

### 3.4. Fluorescence Microscopy Study

Binding of NIRF-CCPM and DG-CCPM to tumor cells was studied using a human epithelial carcinoma A431 cell line. Cells were incubated with NIRF-CCPM (1.54 × 10^15^ particles/mL) and DG-CCPM (3.4 × 10^14^ particles/mL) at 37 °C on cover slips in a 24-well plate (Becton Dickinson Labware, Franklin Lakes, NJ, USA). Each well contained 0.5 ml of Dulbecco’s modified Eagle’s medium/nutrient mixture F-12 Ham (DMEM/F12) containing 10% fetal bovine serum (Gibco, Grand Island, NY). The final concentration of the NIRF-CCPM and DG-CCPM were calculated on the basis of the standard curve. Cells were washed twice with PBS at the end of incubation (2 h or 4 h) and fixed in 95% ethyl alcohol. Data were recorded using a Leica DMR microscope (Leica Microsystems, Bannockburn, IL, USA) equipped with a 75W xenon lamp (750 nm excitation, Chroma Technology, Brattleboro, VT, USA), a Hamamatsu B/W Chilled CCD camera (Hamamatsu Photonics K.K., Hamamatsu City, Japan) and Image-Pro Plus 4.5.1 software (Media Cybernetics, L.P., Silver Spring, MD, USA).

### 3.5. Biodistribution Studies

All animal studies were approved by Institutional Animal Care and Use Committee (IACUC), and were handled and maintained according to IACUC guidelines. The mice were kept under specific pathogen free conditions. A-431 tumor cells were inoculated subcutaneously in the right thigh of nude mice (20–25 g; Harlan Sprague Dawley, Inc., Indianapolis, IN, USA) by injecting 1 × 10^6^ viable tumor cells in suspension in PBS. When tumors had grown to 8–12 mm in average diameter, mice were randomly allocated into 3 groups, with each group consisting of three mice. Mice in group 1 and 2 were injected with ^111^In-DG-CCPM and were euthanized at 4 h and 24 h after injection, respectively. Mice in group 3 were injected with ^111^In-DTPA-NIRF-CCPMand were euthanized at 24 h after injection. Mice in each group were injected intravenously with ^111^In-labeled DG-CCPM at a dose of 1.3 × 10^12^ particles/mouse (10 μCi/mouse). Animals were euthanized by CO_2_ exposure 48 h after injection. Blood, lung, muscle, spleen, kidney, liver, and tumor tissues were removed, weighed, and counted for radioactivity with a Packard Cobra gamma counter (Downers Grove, IL, USA). Uptakes of nanoparticles in various tissues were calculated as the percentage of the injected dose per gram of tissue (%ID/g).

### 3.6. *In Vivo* Gamma Imaging

Nude mice bearing subcutaneous A431 tumors on their upper arm, in two groups of three mice each, were injected intravenously with either ^111^In-DG-CCPM or ^111^In-DTPA-NIRF-CCPMat a dose of 300µCi/4 × 10^13^ particles/mouse. For gamma imaging, mice were placed in the anterior position. Prior to imaging, mice were anesthetized with 2% isoflurane gas (Iso-Thesia, Rockville, NY, USA) in oxygen. During imaging, mice were maintained in an anesthetized state with 0.5%–1.5% isofluranein oxygen. gamma images were acquired using an M-Camgamma camera equipped with medium-energy collimatorand ICON software (Siemens, Hoffman Estates, IL, USA). Images were acquired with the following parameters: matrix: 512 pixels × 512 pixels; zoom: 3.20; energy peak: 247 keV (15%) and 172 keV (15%); total counts: 500 k.

### 3.7. *In Vivo* NIRF Imaging

Whole-body optical imaging was done at various time points up to 120 h after injection using Xenogen—IVIS ® Lumina II imaging system (Hopkinton, MA, USA).The field of view was 12.5 cm in diameter. For *ex vivo* imaging, each organ resected at the end of the imaging study (120 h after injection) were imaged with ICG filter sets (excitation, 745 nm; emission, 810–875 nm). The camera settings included CCD Size 1.3 × 1.3 cm, 1024pixel × 1024pixel resolution, 13 microns pixel size and an exposure time of 1.0 s.

### 3.8. Statistical Analysis

Biodistribution data were analyzed using two-tailed, unpaired Student's t tests, with *p* < 0.05 considered to be statistically significant. The *in vitro* percentage *in vivo* percentage of injected dose per gram (%ID/g) of wet tissue weight, and tumor-to-normal tissue ratios are presented as means ± standard errors of the means. All statistical computations were processed by using a computer software program (Excel; Microsoft, Redmond, WA, USA).

## 4. Conclusions

We successfully synthesized novel glyco-nanoparticles DG-CCPM.It was labeled with ^111^In through radiometal chelator diethylentriaminepentaaceticacid (DTPA) with high radiolableing efficiency. There were no significant differences with respect to tumor-to-normal tissue uptake ratios between ^111^In-DG-CCPM and its corresponding control ^111^In-DTPA-NIRF-CCPM at 4 h and 24 h post injection. However, both nuclear imaging and optical imaging delineated tumor only in mice injected with ^111^In-DG-CCPM at later time points (24–72 h post injection). Further studies to compare the pharmacokinetics and to validate specific tumor uptake of ^111^In-DG-CCPM are needed in order to understand the behaviors of ligand-conjugated CCPM as a platform for targeted delivery of nanoparticles for dual nuclear/NIRF fluorescence imaging. 
